# A case of occult intrahepatic cholangiocarcinoma diagnosed by autopsy

**DOI:** 10.1186/s40792-015-0106-5

**Published:** 2015-10-14

**Authors:** Eri Oda, Daisuke Hashimoto, Yuko Shiomi, Koji Ohnishi, Hiromitsu Hayashi, Akira Chikamoto, Motohiro Takeya, Hideo Baba

**Affiliations:** Department of Gastroenterological Surgery, Graduate School of Medical Sciences, Kumamoto University, 1-1-1 Honjo, Kumamoto, 860-8556 Japan; Division of Surgical Pathology, Graduate School of Medical Sciences, Kumamoto University, 1-1-1 Honjo, Kumamoto, 860-8556 Japan; Department of Cell Pathology, Graduate School of Medical Sciences, Kumamoto University, 1-1-1 Honjo, Kumamoto, 860-8556 Japan

**Keywords:** Cancer of unknown primary, Intrahepatic cholangiocarcinoma, Autopsy

## Abstract

Cancer of unknown primary is associated with unknown biology and dismal prognosis. The most common primary sites of cancer of unknown primary were usually the lungs in autopsy studies, and intrahepatic cholangiocarcinoma is rare. We describe the case of a 57-year-old male patient with systemic lymph node metastasis. Imaging examination failed to reveal primary cancer; however, immunostaining of cytokeratins 7, 19, and 20 of a metastatic axillary lymph node suggested a pancreaticobiliary cancer as a primary lesion. He died of liver abscess and sepsis, and then, autopsy indicated occult intrahepatic cholangiocarcinoma. We discuss the clinical course of this rare cholangiocarcinoma including the diagnostic procedure and also present a review of the English literature regarding patients with cancer of unknown primary.

## Background

Carcinomas of unknown primary (CUP) represent a group of heterogeneous tumors that has no identifiable origin [1]. Despite advances in tumor pathology and imaging techniques, such as positron emission tomography (PET), CUP account for about 5 % of all cancers [2–4] and are associated with a dismal prognosis [5–8]. In such CUP cases, an autopsy is performed to find the primary site.

In this report, we describe the case of a 59-year-old male patient with CUP. The patient was diagnosed with occult intrahepatic cholangiocarcinoma by autopsy. We present a review of the English literature regarding patients with cancer of unknown primary and discuss the clinical course and diagnostic examination for this occult cholangiocarcinoma case.

## Case presentation

**Fig. 1:**
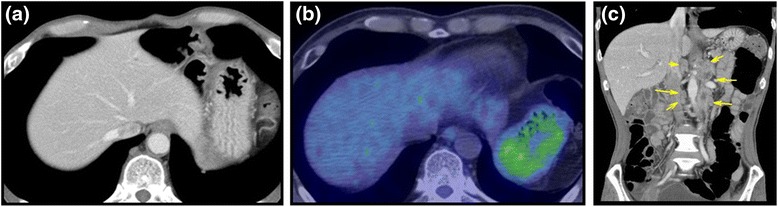
Enhanced CT and PET-CT. CT (**a**) and PET-CT (**b**) failed to detect the tumor in the liver. CT revealed multiple swollen abdominal lymph nodes (*arrows*) (**c**)

**Fig. 2:**
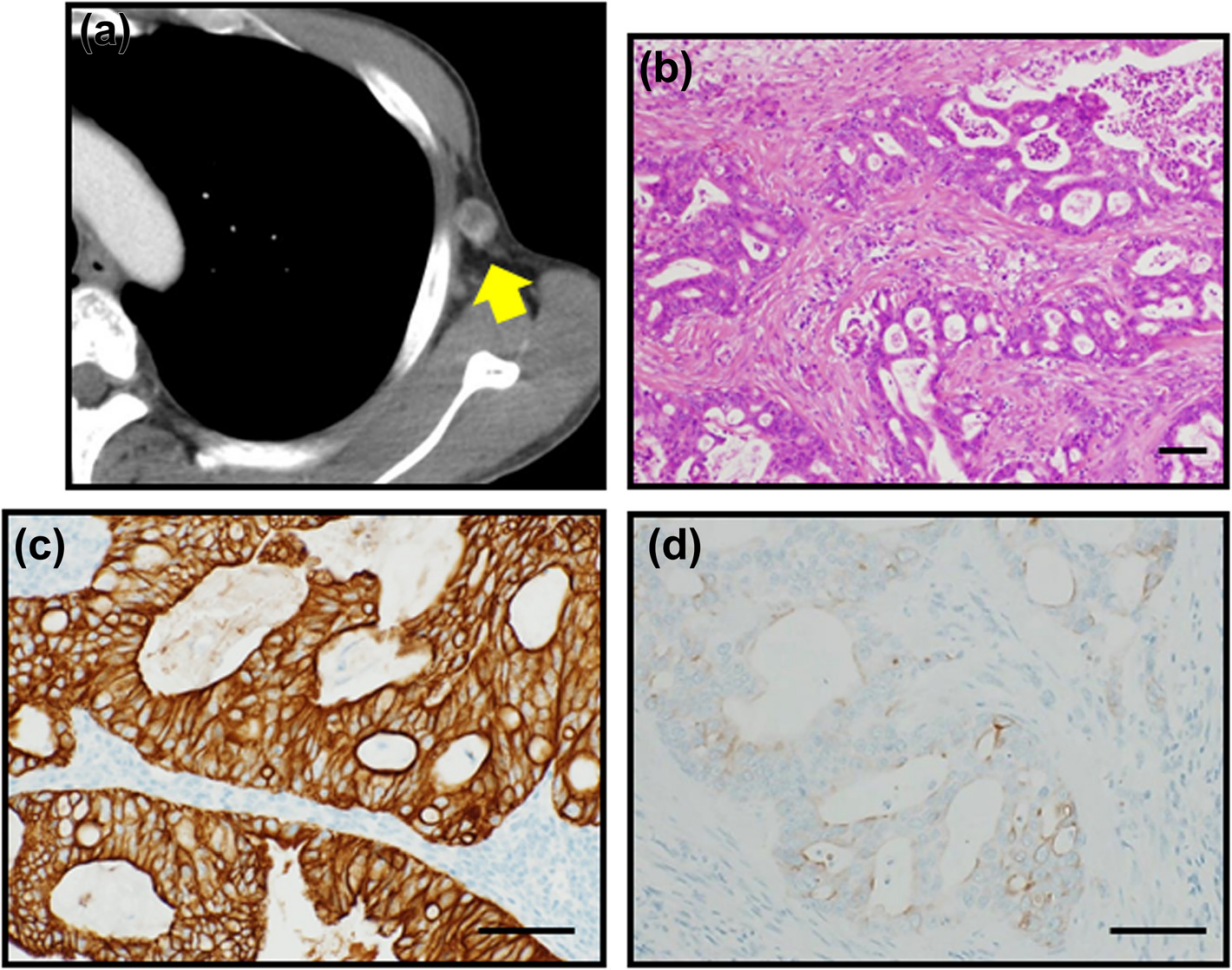
An excisional biopsy of axillary lymph node. CT (**a**) detected a swollen axillary lymph node (*arrow*) and an excisional biopsy was performed. The histological diagnosis of the lymph node was a metastasis of adenocarcinoma (**b**). Immunohistochemistry for CK7 was positive (**c**) and that for CK20 was almost negative (**d**). *Bar* 10 μm

A 57-year-old male was investigated because of elevation of tumor markers (carcinoembryonic antigen (CEA) 12.9 mg/ml, carbohydrate antigen 19-9 (CA19-9) 658.5 U/ml). Enhanced computed tomography (CT) (Fig. 1a) and PET-CT (Fig. 1b) and endoscopy failed to detect a suspected primary lesion. As CT revealed multiple swollen abdominal (Fig. 1c) and axillary lymph nodes (Fig. 2a), an excisional biopsy of an axillary lymph node was performed. The histological diagnosis of the lymph node was a metastasis of adenocarcinoma (Fig. 2b). Because immunohistochemistry of the lymph node for cytokeratin (CK) 7 (Fig. 2c) and CK19 was positive and that for CK20 was almost negative (Fig. 2d), pancreaticobiliary cancer was suspected as primary lesion. Then, endoscopic retrograde cholangiopancreatography (ERCP) was performed; nevertheless, the primary lesion was not discovered. Biopsy from epithelium of the bile duct was obtained during ERCP, and the malignant cell was not found. Combination chemotherapy of gemcitabine and cisplatin was introduced; however, his disease had progressed. The patient died of liver abscess and sepsis 10 months after the introduction of chemotherapy. All diagnostic modalities which the patient underwent to obtain a diagnosis are listed in Table 1.

**Table 1:** Diagnostic modalities which the patient underwent to obtain a diagnosis

Examination	Findings
Tumor marker	CEA 12.9 mg/ml
	CA19-9 658.5 U/ml
Gastrointestinal and colorectal endoscopy	No significant findings
CT	Multiple swollen abdominal and axillary lymph nodes
PET-CT	Multiple swollen abdominal and axillary lymph nodes without abnormal uptake
Immunohistochemistry of the lymph node	CK7 and CK19 were positive
	CK20 was almost negative
ERCP	No significant findings
Biopsy from epithelium of the bile duct	No malignancy

**Fig. 3:**
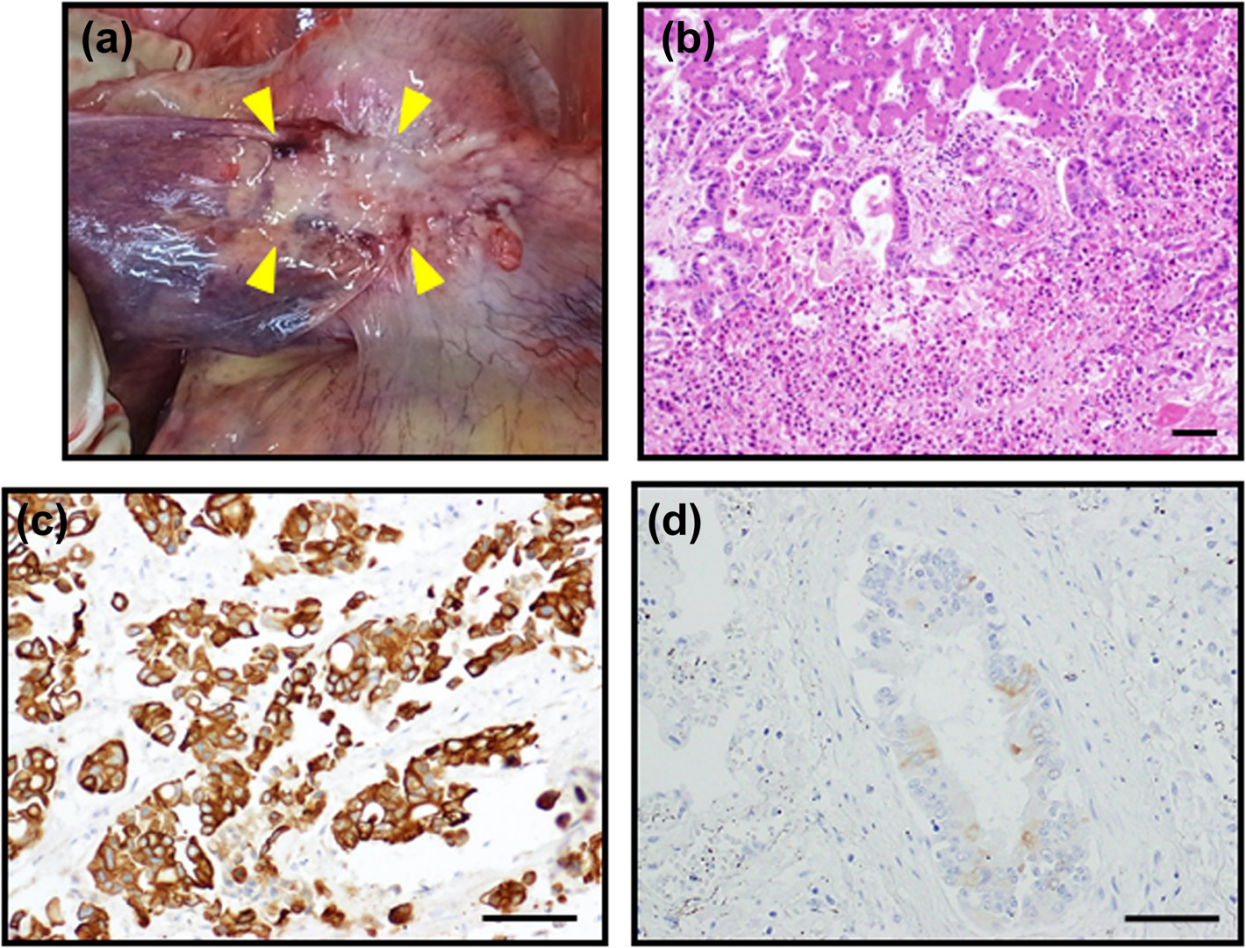
Postmortem findings. Macroscopically, a solid tumor (*arrowheads*) in the lateral segment of the liver was discovered (**a**). At histology, intrahepatic cholangiocarcinoma was observed (**b**). Immunohistochemistry of the lymph node for CK7 (**c**) and CK20 (**d**) was similar. *Bar* 10 μm

Autopsy was performed to find the primary lesion. Macroscopically, a gray-white colored, ill-defined solid tumor in the lateral segment of the liver was found, invading the diaphragm (Fig. 3a). Pathological diagnosis was intrahepatic cholangiocarcinoma (Fig. 3b). Immunohistochemistry revealed that these tumor cells were positive for CK7 (Fig. 3c) and CK19 and were negative for CK20 (Fig. 3d), as well as axillary lymph node metastasis.

We reviewed recent English literature regarding patients with CUP [9–12] (Table 2). The most common pathology of CUP was adenocarcinoma, and the most common primary sites found by autopsy were usually the lungs followed by the pancreas. The possible reason why the lung is the common primary site in CUP is that small cell carcinoma is likely to develop metastasis even in its early stages [13]. However, we are not aware of similar cases with intrahepatic cholangiocarcinoma. The advantages of an autopsy in such cases are to identify the primary site, to provide closure for family members, and to correlate findings with antemortem investigations [9, 14, 15], in spite of the damaging disadvantage of the body. Autopsy can still play an important role, especially the problem-oriented autopsy in which a clinician provides clinical diagnoses and raises a specific question to be answered by the pathologist, like the present case [16, 17].

**Table 2:** Recent literature summary of studies of patients with cancer of unknown primary

Author	Total number of patients	Common pathology (no.)	Autopsy cases	Primary site identified	Common primary site (no.)
Blaszyk [10]	64	Adenocarcinoma (51), squamous carcinoma (3)	64	35	Pancreas (13), intestine (11), lung (8), ovaries (1), prostate (1)
Mayordomo [11]	43	Adenocarcinoma (23), undifferentiated (4), squamous carcinoma (3)	43	35	Bile duct (7), pancreas (6), lung (4), prostate (3), stomach (2)
Maiche [12]	109	Adenocarcinoma (37), squamous carcinoma (33), undifferentiated (31)	64	43	Lung (13), kidney (6), pancreas (4), intestine (4), liver (3)
Al-Brahim [9]	53	Adenocarcinoma (37), undifferentiated (5)	53	27	Lung (7), pancreas (4), stomach (3), bile duct (1), appendix (1)

The reason why we failed to detect this intrahepatic cholangiocarcinoma using many imaging modalities is considered as follows. Because cardiac pulsation can interfere with diagnostic imaging, it may be difficult to detect the solid tumor in the subphrenic area of the lateral segment of the liver. This area should be considered as one of the blind spots of imaging examination. There were no abnormal findings which could indicate the existence of the cancer lesion from the retrospective viewpoints. If an exploratory laparoscopy was performed, we might have found this intrahepatic cholangiocarcinoma. The result of the immunohistochemistry of the axillary lymph node was accurate in this case, so the treatment choice of chemotherapy with gemcitabine and cisplatin was adequate.

## Conclusions

Despite advances in diagnostic imaging technology, identifying the primary sites in patients with metastatic malignancies is sometimes difficult even now. In the presented case, immunohistochemistry was accurate and useful, and exploratory laparoscopy may play a significant role to detect the primary lesion. Thus, various examinations should be performed for CUP patients to receive sufficient treatment.

## Consent

Written informed consent was obtained from the patient for publication of this case report and any accompanying images. A copy of the written consent is available for review by the Editor-in-Chief of this journal.

## Abbreviations

CA19-9: carbohydrate antigen 19-9
CEA: carcinoembryonic antigen
ERCP: endoscopic retrograde cholangiopancreatography
CT: computed tomography
CUP: carcinomas of unknown primary
PET: positron emission tomography
